# Organized Hematoma of the Maxillary Sinus: Report of Two Cases

**DOI:** 10.22038/ijorl.2021.48295.2594

**Published:** 2021-05

**Authors:** Mohamed Amin Chaabouni, Souha Kallel, Rania Kharrat, Ines Kharrat, Manel Mellouli, Bouthaina Hammami, Ilhem Charfeddine

**Affiliations:** 1 *Department of Otolaryngology–Head and Neck Surgery, Habib Bourguiba Hospital, Sfax , Tunisia University of Sfax.*; 2 *Department of Pathology, Habib Bourguiba University Hospital, Sfax, Tunisia.*

**Keywords:** Epistaxis, Endonasal endoscopic surgery, Maxillary sinus, Nasal obstruction, Organized hematoma, Sinonasal tumor

## Abstract

**Introduction:**

Organized hematoma of the maxillary sinus (OHMS) is a rare benign disease that can be locally aggressive. The diagnosis of this condition is challenging.

**Case Reports:**

We report two cases of OHMS presented with recurrent nasal bleeding, nasal obstruction and anosmia. Radiological findings were suggestive of a vascularised lesion in the first case and a malignant tumor of the maxillary sinus in the second case. Both patients underwent an endonasal endoscopic surgery, There was no recurrence at 19 months’ and six months’ follow-up respectively.

**Conclusions:**

OHMS should be included in the differential diagnosis if a patient presents with history of recurrent epistaxis and nasal obstruction and radiological findings reveal an expansible maxillary mass with or without bone erosion. Correct preoperative diagnosis is important to avoid unnecessary extensive surgery. The prognosis is very good and minimally invasive surgery such as endonasal endoscopic surgery can cure it completely.

## Introduction

Sinonasal organized hematoma (OH) is a rare benign lesion. However, it can be locally aggressive and accompanied by bone destruction (1). It presents as a progressively enlarging mass (2). These characteristics lead to misdiagnoses of malignant tumors or other locally aggressive diseases such as inverted papilloma, fungal rhinosinusitis, odontogenic tumors or mucocele (1,2). To differentiate the OH from a malignant tumor, the complete absence of neoplastic cells in the totally removed specimen should be confirmed (3). This lesion mainly arises from the maxillary sinus and is known as organized hematoma of maxillary sinus (OHMS). It may be also located in the nasal cavity, sphenoid sinus, or frontal sinus (4). Its exact pathogenesis remains unclear. The clinical and radiologic characteristics of organised hematoma are not well known and not specific and the diagnosis still challenging (5). The treatment is based on complete surgical removal of the lesion more often via endonasal endoscopic surgery (EES) (6).In this paper, we report two cases of OHMS and we discuss the pathogenesis and the diagnostic and therapeutic features of this uncommon lesion.

## Case Reports

Case 1

**Fig 1:**
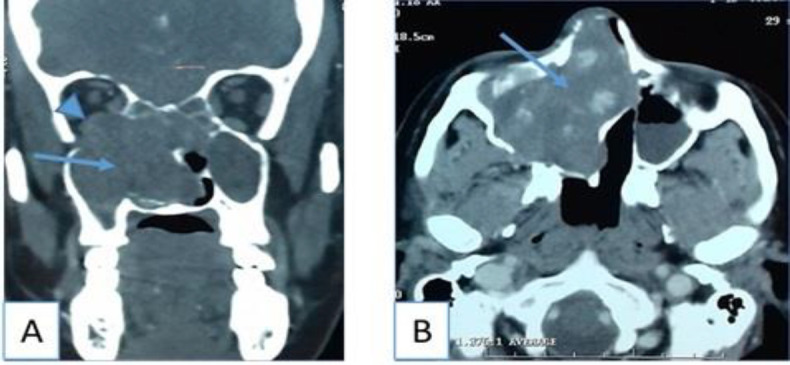
CT of the paranasal sinuses: (A) nonenhanced coronal section shows heterogenous soft-tissue mass (arrow) in the right maxillary sinus, the ethmoid and the nasal cavity with destruction of the medial wall of the sinus and the nasal septum, destruction of the orbital floor and extension of the mass into the peri-orbita (arrow head). (B) enhanced axial section shows nonhomogeneous enhancement

A previously healthy 47-year-old woman presented to the emergency with abundant epistaxis. She had a prior history of recurrent nasal bleeding, rhinorrhea, right-sided nasal obstruction and anosmia for one month. She had no significant medical problem including bleeding diathesis, facial trauma, or sinus surgery. Epistaxis was treated with nasal packing, which was removed two days after. Anterior rhinoscopic evaluation demonstrated a reddish mass that bled easily occupying the right nasal cavity. The right lateral nasal wall was medially displaced towards the nasal septum, which was also deviated to the left side. Computed tomography (CT) of the paranasal sinuses revealed a 6,5×4,5 cm heterogeneous soft-tissue mass in the right maxillary sinus and the ethmoid with associated destruction of the medial wall of the sinus, extension to the nasal cavity and destruction of the nasal septum. Destruction of the right orbital floor and extension of the mass into the peri-orbita were also noted. Contrast enhanced scan revealed a nonhomogeneous mass with important contrast enhancement (Figure 1). 

**Fig 2:**
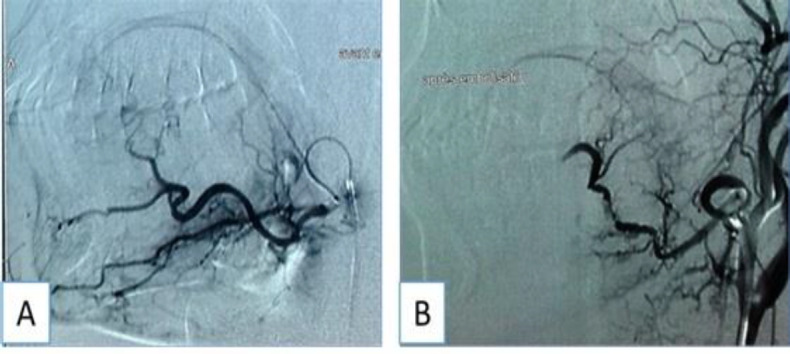
Arteriography before (A) and after (B) embolization

Since the clinical and radiological findings raised the index of suspicion for a vascularised lesion and the risk of profuse bleeding an angiography was performed (Figure 2). 

**Fig 3:**
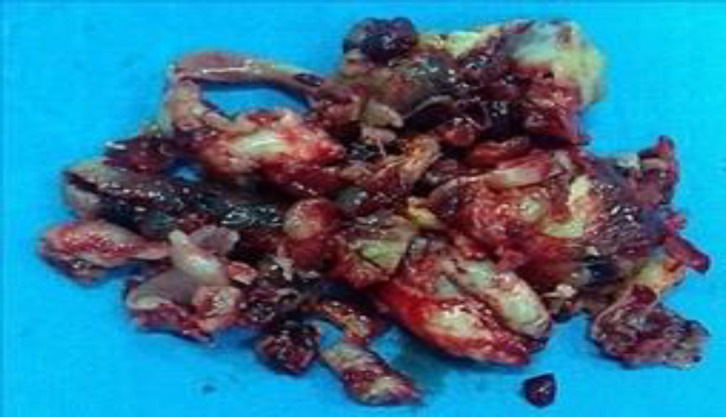
Specimen of organized hematoma: friable dark red portions and fibrous portions (color should be used)

It showed a strongly enhanced lesion and found that facial artery was the feeding artery. Embolization was successfully performed. The patient underwent EES. Although the mass was friable, complete resection was achieved (Figure 3).

Bleeding was not serious during surgery. There were no postoperative complications. Histopathological finding was consistent with a fibrous encapsulated organized hematoma without neoplastic tissue. There was no recurrence of lesion at 19 months’ follow-up.

Case 2

**Fig 4:**
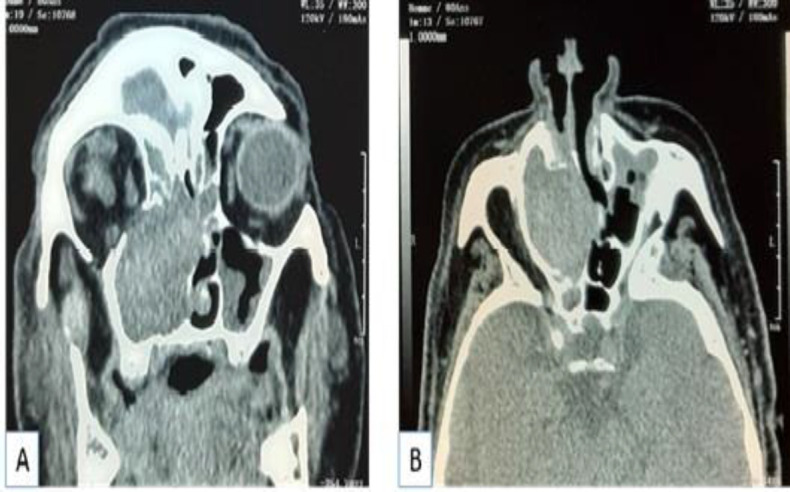
Nonenhanced CT of the paranasal sinuses: coronal section (A) and axial section (B) show a well-defined expansible mass of the right maxillary sinus, the frontal sinus, the ethmoid and the nasal cavity with destruction of the medial wall of the sinus, the nasal septum and the orbital floor.

An 80-year-old man presented with a three years’ history of recurrent epistaxis from right nasal cavity, right nasal obstruction and anosmia. He already had diabetes and dyslipidemia. This patient had no past history of nasal or paranasal sinus surgery or trauma and did not receive anticoagulant drugs. On physical examination, no facial deformity was detected. Endoscopic examination revealed a friable reddish mass at the right middle meatus and a polyp occupying the right nasal cavity. Nasal septum was deviated to the left side. CT showed a well-defined expansible mass of the right maxillary sinus, the frontal sinus, the ethmoid and the nasal cavity with bone destruction (Figure 4). 

**Fig 5:**
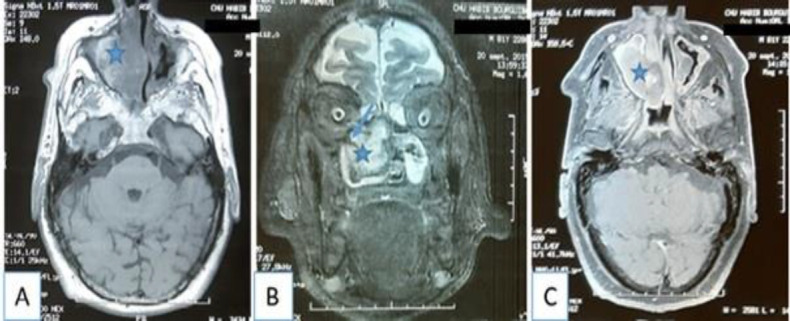
MRI of the parasansal sinuses: (A) axial section of T1-weighted: a well-defined expansible mass of the right maxillary sinus and the nasal cavity with low signal intensity. (B) coronal section of T2-weighted: the mass demonstrates heterogeneous high signal intensity with extension into the right orbit (arrow). (C) axial section of contrast-enhanced MRI: heterogeneous enhancement of the mass

**Fig 6:**
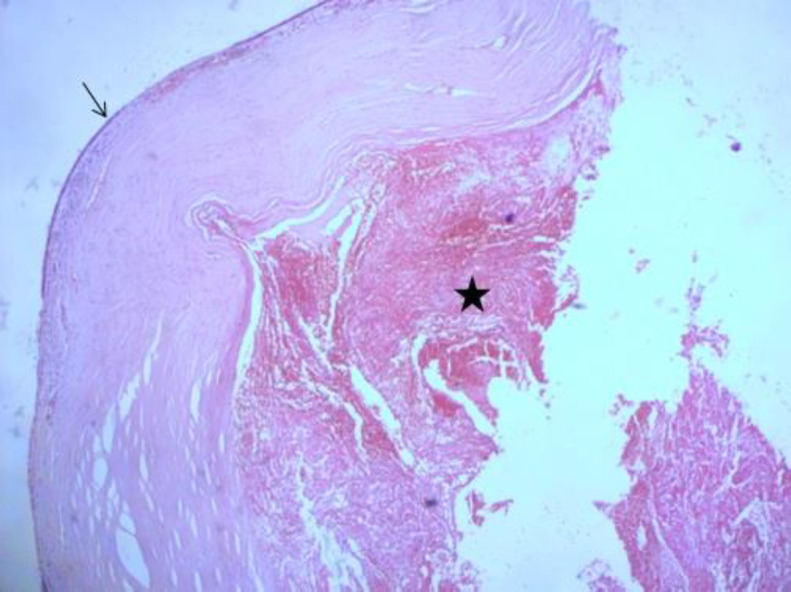
Sinonasal organized hematoma: the respiratory epithelium (arrow) is lifted by a cystic cavity (asterix) containing hemorrhage and fibrin. The cyst wall is consisting of hyalinized, hypocellular fibrous tissue

On magnetic resonance imaging (MRI) (Figure 5), the masse was well demarcated from the surrounding structures and showed low signal intensity on T1-weighted imaging and demonstrated heterogeneous high signal intensity on T2-weighted imaging with extension into the right orbit. On contrast-enhanced MRI, heterogeneous enhancement was observed. The size of the mass was 32 × 39 mm. MRI findings were suspicious for a malignant tumor of the maxillary sinus. Preoperative biopsy was performed and the pathologic examination of this specimen demonstrated an inflammatory polyp. Endonasal endoscopic surgery was performed to resect the lesion. Mass was completely removed using middle meatal antrostomy and the mucosa of the maxillary sinus was found as intact and normal appearance. The postoperative course of the patient was uneventful. Histologically, the specimen consisted of old hematoma with hemosiderin pigment and fibrous tissue with fibrin material, fibroblast and neovascularization, which was consistent with an organized hematoma, without evidence of neoplasm (Figure 6). There was no recurrence at 6 months’ follow-up.

## Discussion

OHMS is a rare nonneoplastic but locally invasive lesion (2). The exact pathogenesis is not fully elucidated. Repeated hemorrhage in a semi-closed lumen is a possible theory that has been suggested. OHMS appears in patients with and without a bleeding diathesis (7). Although not present in our patients, various factors may cause hemorrhage, such as facial trauma, postoperative bleeding, vascular disease and vessel injury (8). The association of OH with bleeding tendency, such as coagulation factor deficiency or end stage renal failure has been described in the literature (9).

Regardless of the initial process of the hematoma, poor ventilation and drainage in the maxillary sinus impede resorption of the hematoma and results in neovascularization and fibrosis (4). It has been suggested that persistent negative pressure in a semi-closed cavity following the initial episodic hemorrhage, promotes repeated rupturing of fragile mucosal vessels and neovascularized vessels, which in turn lead to progressive enlargement of the mass and erosion of the adjacent bony structures (10).

The most common clinical manifestations, which were also present in our cases, are nasal obstruction and repeated epistaxis (1). Other symptoms may be present, such as cheek pain, facial swelling, headache, rhinorrhea, and hyposmia (7,11). Endonasal examination demonstrates nonspecific features such as nasal polyp, bloody discharge, medial displacement of the lateral nasal wall or a mass in the nasal cavity (1). Facial paresthesias, retro-orbital pain, orbital displacement and/or proptosis may be present in case with orbital invasion (2).

A correct preoperative diagnosis is important for determining a therapeutic strategy and avoid unnecessary extensive surgery. CT and MRI are strongly recommended however radiologic appearances are nonspecific and differential diagnosis from neoplastic pathology is problematic (6). CT findings include an expansive, well demarcated and compressive mass with bone erosion of the medial sinus wall and the uncinate process (10,12). After contrast administration, patchy heterogeneous enhancement is usually observed (1). A papillary or frond-like enhancement pattern is a characteristic feature of OH (8). However, CT alone does not furnish enough information and MRI can be helpful to differentiate OH from locally aggressive neoplasms and to determine the extent of the lesion (3,5). Typically, OH exhibits heterogeneous signal intensities in T1 and T2 weighted imaging (3). The central part of the lesion presents low to intermediate signal intensity on T1-weighted imaging and high signal intensity on T2-weighted imaging (5,13). The peripheral fibrous capsule has an hypointense signal on T2-weighted images (8). After contrast administration, discrete areas of enhancement in a patchy distribution are present (7). 

The mucosa is well enhanced on T1-weighted images with contrast and has high intensity on T2-weighted images, which suggests the inflammatory change and differs from a malignant tumor that causes mucosal invasion (14). Park et al presented 18FDG-PET findings of OH that showed a moderately increased FDG uptake in the periphery of the mass with central photon defect (5).

Treatment strategy is based on complete surgical excision. Surgical approach and extent of the resection depend on size of the lesion and the involvement of adjacent compartments (4). Previously, patients have undergone the Caldwell-Luc approach or a lateral rhinotomy incision (15). With the development of endoscopic surgical techniques and instruments, endoscopic surgery was described as a valid and effective surgical approach to treat this disease (6,13). 

Preoperative biopsy has no serious complication but does not provide helpful information in differentiating malignant processes (12). Embolization can be performed before surgery, if the feeding artery is found with angiography, to decrease bleeding volume (14). If it is present, a bleeding disorder must be well controlled to prevent excessive intraoperative bleeding and OH recurrence (2,8). Sinonasal organized hematoma is confirmed on histopathology. Microscopic examination reveals dilated vessels, hemorrhage, necrosis, fibrosis, hyalinization, and neovascularization with no evidence of malignancy (8)

The prognosis of OH after complete surgical removal is usually very good and it rarely recurs(3).

## Conclusion

OHMS is a rare benign lesion that may mimic a neoplasm or other benign aggressive pathology. If a patient presents with a history of frequent epistaxis and nasal obstruction and radiological findings reveal an expansile maxillary mass with or without bone erosion, OHMS should be included in the differential diagnosis. The prognosis is very good and minimally invasive surgery such as endonasal endoscopic surgery can cure it completely.
